# Association between pain sensitivity and distance from the affected level of disc herniation

**DOI:** 10.1038/s41598-025-24918-4

**Published:** 2025-11-28

**Authors:** Johanne Brinch Filtenborg, Andrew Haig, Berit Schiøttz-Christensen, Kirstine Amris, Gilles Ludger Fournier, Rune Mygind Mieritz, Dorthe Schøler Ziegler, Søren O’Neill

**Affiliations:** 1 Medical Spinal Research Unit, Spine Centre of Southern Denmark, University Hospital of Southern Denmark, Kolding, Denmark; 2 Department of Regional Health Research, Faculty of Health Services, University of Southern Denmark, Odense, Denmark; 3 The Parker Institute, Bispebjerg and Frederiksberg Hospital, University of Copenhagen, Copenhagen, Denmark; 4 The University of Michigan, Ann Arbor MI, 48109 USA; 5 Department of Rheumatology, Bispebjerg and Frederiksberg Hospital, Copenhagen, Denmark; 6 Department of Neurosurgery, University Hospital Odense, Odense, Denmark; 7 Department of Clinical Research, Faculty of Health Sciences, University of Southern Denmark, Odense, Denmark; 8 Haig Physical Medicine, PLC, Vermont 05753 Middlebury, USA; 9 Research Unit of General Practice, University of Southern Denmark, Odense, Denmark

**Keywords:** Anatomy, Diseases, Health care, Medical research

## Abstract

A frequent cause of acute low back pain with radiating leg pain is lumbar disc herniation. In a clinical context, it can be very difficult to know which segment, let alone which anatomical structure, is responsible for the nociception that patients perceive as low back pain. Quantitative sensory pain testing (QST) is a method that quantifies the pain response. The aim of this study was to investigate whether the level of disc herniation correlates with mechanical and/or thermal pain thresholds in patients with lumbar disc herniation, with the lowest pain thresholds being at the affected level. A cross-sectional study with 102 participants with lumbar disc herniation verified by MRI was conducted. Participants were tested at baseline with an algometer measuring pressure pain threshold (PPT) and a thermode measuring heat pain threshold (HPT). Measurements were collected at five sites, 2 cm lateral to the L1 to L5 processus spinosi. The data showed a significant association between the proximity to the level of disc herniation to the QST test site and the mechanical PPT thresholds with the lowest pain thresholds closest to the level of disc herniation (slope, 6.1; 95%CI 1.79 to 10.4; *p* = 0.005). There was no association for HPTs. Pain thresholds were associated with the level of disc herniation when measured with mechanical pressure but not with heat.

**Supplementary Information:**

The online version contains supplementary material available at 10.1038/s41598-025-24918-4.

## Introduction

 A frequent cause of acute low back pain with radiating leg pain is lumbar disc herniation (LDH). The clinical diagnosis of radiculitis or radiculopathy is based on clinical history and physical examination, including neurological assessment. The diagnosis is typically confirmed by Magnetic Resonance Imaging (MRI) as recommended^1–3^.

Often, patients will present with several degenerative changes on MRI, including multiple disk herniations and protrusion, making it difficult to determine which of them are of relevance to the clinical presentation^4^. Furthermore, such degenerative disc changes are also frequently found on MRI in asymptomatic individuals^5–8^. Also it is well known that anatomical variations in innervation may further obfuscate the relationship between MRI findings and clinical neurological findings^9^.

In terms of pain location, a number of different studies have demonstrated considerable overlap in pain distribution with pain elicited from the intervertebral discs, facet joints, sacroiliac joints (SI) and hip joints, and lumbo-pelvic muscles^10^. Consequently, in a clinical context, it can be very difficult to know with certainty which segment, let alone which anatomical structure is responsible for the nociception that patients perceive as low back pain and/or radiating leg pain.

Quantitative sensory testing (QST) of pain sensitivity covers a number of well-defined psychophysical methods that standardize a potentially painful stimulus and quantifies the pain response of the test subject, making it possible to investigate pain processing^11^.

Whereas the value of QST as a clinical tool is debated^12^, it is increasingly being recognized that disturbances in pain processing and ensuing increased pain sensitivity is an important factor in the clinical course of many painful conditions. Standardized assessment of pain sensitivity potentially could provide important clinical information. This is probably the case for both localized and generalized changes in pain sensitivity^13^. Whilst it is well known that secondary hyperalgesia, i.e. increased pain sensitivity in local tissues adjacent to the source of noceiception, is a common phenomenon, it has to our knowledge not yet been studied, whether pain sensitivity is different at the level of disc herniation, compared to other spinal segments.

The aim of this study was to test the hypothesis that the level of disc herniation correlates with paraspinal mechanical and/or thermal pain thresholds in patients with lumbar disc herniation, with the lowest pain thresholds being at the affected level. The specific objective was to investigate whether patients are sensitized at the level of disc herniation when examined by quantitative sensory testing.

## Methods

### Setting

The data from the present study were part of a prospective, 4-year observational study of individuals with sciatica due to lumbar disc herniation (Clinicaltrials.gov registration number NCT03832036, clinical trial registration start date: October 1 st 2018). The aim and objectives were formulated a-priori, before data collection commenced. All experiments were performed in accordance with relevant guidelines and regulations.

Individuals were consecutively recruited between November 2018 and January 2021 from three different hospitals in Denmark; (a) The Spine Center of Southern Denmark at Lillebaelt University Hospital, (b) The Department of Neurosurgery at Odense University Hospital and (c) The Department of Rheumatology at Frederiksberg Hospital.

Participants were thus all individuals recruited in a hospital setting on referral from private practice.

Study data were collected and managed using REDCap electronic data capture tools hosted at OPEN, Open Patient data Explorative Network, Odense University Hospital, Region of Southern Denmark^14,15^.

## Participants

Participants were identified by the attending clinician and their eligibility to participate was determined on the basis of the following criteria:

- 18 + years of age and legally competent.
- fluent in Danish (written and spoken).
- MRI-verified disc herniation in the lumbar spine.
- primary complaint of low back pain radiating below the knee or anterior thigh in one or both legs.
- pain distribution recognizable as a dermatomal pattern.
- reported average pain intensity of 3 or more on a Numerical Rating Scale (0–10 NRS).
- no history of previous spine surgery, surgery in general in the past 4 months, current use of anticoagulants and/or confounding diagnoses e.g. lumbar spinal stenosis, local muscle trauma, cancer, metastases, fibromyalgia and neuropathy.

Participants eligible to participate who expressed an interest in participation were informed of the study in writing and in person and scheduled for enrollment, once informed consent had been obtained.

## Clinical data

### Patient-reported outcomes

Prior to the QST assessment, participants completed a digital questionnaire. Data were collected on: height (cm), weight (kg), average low-back pain intensity (0–10 NRS), average leg pain intensity (0–10 NRS)^16^ and use of pain medication. A pain drawing was filled out as pen-on-paper. Participants were instructed to indicate the areas of pain by shading or marking the corresponding body parts. This was transformed to a digital PDF, where the total area of pain was calculated as number of pixels^17^.

## Symptomatic level of disc herniation

Data were collected from the participants’ journals and routine MRI scans. The symptomatic level of disc herniation was recorded in the participant’s hospital journal in accordance with the ICD-10. The diagnosis was based on an overall clinical assessment made by the attending clinician at the hospital unit. This reflects real-world clinical decision-making. Participants could only enter the study if they had an MRI that verified the clinical diagnosis made by the clinician. Thus, the diagnosis was clinically and MRI determined. MRI scans for each patient were reviewed by two experienced musculoskeletal radiologists who were blinded to all patient information. In cases of involvement of multiple levels or bilateral nerve involvement, the most affected level or side of intervertebral disc degeneration was chosen as the primary anatomical structure/level generating symptoms. This was based on evaluation of several MRI parameters, including Pfirrmann classification grade I-V, evaluation of disc contour, herniation type and location, nerve root involvement and location. Thus, for each participant one side and symptomatic level of disc herniation was recorded.

### Quantitative sensory testing protocol

The QST assessment was performed by 3 examiners, following a standardized and scripted procedure inspired by The German Research Network on Neuropathic Pain QST protocol^18^. The QST test battery included several pain inducing modalities and measurements, including temporal summation assessed via a mechanical probe and conditioned pain modulation assessed via the cold-pressor test. The results of these QST assessments will be reported in a separate paper. For the current study, we focused on the local segmental measurements in the lumbar region, where PPT and HPT were applied. The examiners underwent extensive training in the QST procedure to ensure they were familiarized with the protocol. Verbal instructions to the participants were recorded digitally and played on an iPad, which ensured the exact wording in the instructions given to the participants (see Supplementary Appendix).

Initially, 5 test-sites were marked with a black felt-tip pen at the level of the spinous processes of L1 to L5, based on palpatory findings^19^, 2 cm lateral from the midline on the most painful side (right/left) as identified by the participant. This was done by an experienced clinican skilled in palpation.

The thermal and mechanical QST tests were performed twice at each of these five test-sites. Two series of five pressure pain threshold tests, one for each segment, were performed in random order, followed by two series of five thermal pain thresholds in random order. The average of the two measurements at each test-site was used for further analysis. Randomization was done using an a-priori computer-generated test order for each series of five tests, for each participant.

## Pressure pain threshold

Pressure was applied manually with a Somedic algometer type II (Hörby, Sweden, 1 cm2 probe) perpendicular to the skin on the marked test-sites, with a near-constant increase in pressure of 50 kPa/s until the participant indicated the pressure becoming painful by pressing an indicator button connected to the algometer.

## Thermal pain threshold

The heat pain threshold was measured at each test site with a Medoc TSA-II thermode stimulator. The size of the thermode was 30 × 30 mm. The thermode stimulator had a baseline temperature of 32 °C and increased 1 °C/s. The participant indicated when the stimulus was perceived as becoming painful by pressing an indicator button, upon which the temperature returned to baseline (decrease 10 °C/s).

### Statistical methods

We enlisted the help of a bio-statistician from the Department of Regional Health Service Research, University of Southern Denmark. The sample size was dictated by a logitudinal analysis on data from the same cohort to be published separately.

Baseline characteristics are presented with descriptive statistics, including means with standard deviation (SD) when normally distributed, otherwise as median with interquartile range. Dichotomous and categorical data are presented in proportions and/or frequency/count. Normality of the data was assessed using Q-Q plots and histograms.

Linear mixed models were used to investigate the association between pain sensitivity and the distance in number of segments between the symptomatic level of disc herniation and test site of QST of pressure pain threshold and heat pain threshold. In the model the symptomatic level of disc herniation is treated as a categorical variable, with each segment representing a distinct level (e.g., L1, L2, L3, L4, L5). Values reported are least-squares means with 95%CIs and the models include the participant as random effect, proximity to symptomatic disc herniation (5 levels; 0, 1, 2, 3, 4 segments) as fixed effect, as well as the value at the level 0 segments as a covariate. Missing data for a specific distance were handled implicitly by the repeated measures mixed linear models, assuming data missing at random (MAR).

Assumptions were checked by visual inspection of residual plots assessing the normality of residuals. Additional analyses were stratified by site of symptomatic disc herniation (5 levels; L1, L2, L3, L4, L5) and based on a similar model.

The analyses were pre-specified in a statistical analysis plan before performing any analyses (Supplementary File). The analyses were performed using R v.4.4.2 (R Core Team (2022), R Foundation for Statistical Computing, Vienna, Austria) with the package lme4^20^ and emmeans^21^.

### Ethics

The study was approved by the Local Ethics Committee (Region of Southern Denmark, approval no: S-20170138).

## Results

### Participants

A total of 16,445 patient visits were recorded in the period of inclusion (November 2018 to January 2021), which included patients diagnosed with a lumbar disc herniation with/without radiculopathy seen at the three hospitals (The Spine Center of Southern Denmark at Lillebaelt University Hospital *n* = 8,984, The Department of Neurosurgery at Odense University Hospital *n* = 1,870 and The Department of Rheumatology at Frederiksberg Hospital *n* = 5,591).

**Fig. 1:**
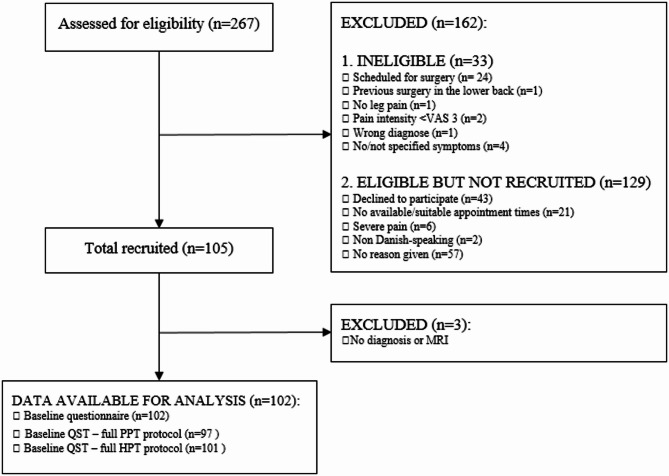
Flow chart of participant inclusion.

267 individuals were assessed for eligibility and 105 individuals were enrolled in the study – see Fig. 1. Main reasons for exclusion were individuals being scheduled for early surgery and therefore not meeting the inclusion criteria, individuals declining to participate and individuals not being able to participate on the scheduled dates for assessment.

Of the 105 participants included, complete baseline data were available for 102 participants. 3 participants had missing data on their diagnosis and were excluded from further analyses.

Five participants had missing data on the PPT outcome at baseline and 1 participant had missing data on the HPT outcome at baseline – these participants were retained in the analysis, with missing data handled using appropriate statistical methods.

### Descriptive statistics

Just under half of the participants were female. The mean age was 48 (SD = 14) and the average Body Mass Index was 27 (SD = 5). The mean LBP intensity (0–10 NRS) was 4 (SD = 2) and the mean leg pain intensity (0–10 NRS) was 5 (SD = 3). 3/4 of the participants had reported previous episodes of back pain or leg pain (ischias). 67% were using pain medication, among those the majority had used pain medication for more than 2 months.

On the pain drawings, the average area of pain was 5,144 (SD = 4,631). The areas of the pre-defined anatomical regions are as follows: the lower back (637 pixels), buttock (1,760 pixels) and thigh and calf (5,460 pixels)^17^.

53 participants (52%) were reported to have a disc herniation at the level of L5/S1, 38 participants (37%) at the level of L4/L5, 5 participants (4.9%) at the level of L3/L4, 5 participants (4.9%) at the level of L2/L3 and 1 participant (1%) at the level of L1/L2.

Demographic and clinical characteristics of the participants are shown in Table 1.

**Table 1:** Participant Characteristics. The work status category “employed” consists of participants who have answered either “Ordinary job – full time or part-time”, “Flexjob”, “Studying” or “Undertaking rehabilitation”. The category “unemployed” consists of participants who have answered either “Unemployed”, “Receiving disability or retirement pension”, “Housewife or house husband”, “Other”, “Don’t want to answer” or “Don’t know”.

Characteristic	*N* = 102^*1*^
Age, years	47.56 (13.95)
Sex	
Female	48 (47%)
Male	54 (53%)
Body Mass Index, kg/m^2	26.63 (4.81)
Back pain, NRS (0–10)	4.40 (2.21)
Leg pain, NRS (0–10)	5.13 (2.18)
Previous pain	
Yes	78 (77%)
No	22 (22%)
Don’t know	1 (1%)
Use of pain medication	68 (67%)
Duration of pain medication	
Less than 1 week	2 (3%)
Between 1 week and 2 months	19 (28%)
More than 2 months	46 (68%)
Don’t know	1 (1%)
Physical activity	
Inactive	14 (14%)
Low-impact physical activity ≥ 4 h/week	59 (58%)
High-impact physical activity ≥ 3 h/week	24 (24%)
Don’t know	4 (4%)
Smoking status	
No, I don’t smoke cigarettes	72 (71%)
Yes, I smoke cigarettes only occasionally	13 (13%)
Yes, I smoke 1 to 4 cigarettes per day	1 (1%)
Yes, I smoke 5 to 14 cigarettes per day	11 (11%)
Yes - I smoke 15 to 24 cigarettes per day	3 (3%)
I smoke 25 or more cigarettes per day	0 (0%)
Don’t know	1 (1%)
Alcohol status (units per week)	
0	35 (35%)
1–7	47 (47%)
8–14	14 (14%)
15 or more	5 (5%)
Don’t know	0 (0%)
Work status	
Employed	73 (72%)
Unemployed	28 (28%)
Sick leave	
Yes	34 (34%)
No	67 (66%)
Don’t want to answer	0 (0%)
Roland-Morris Questionnaire	53.33 (24.14)
EQ5D-3L, index score	0.65 (0.16)
EQ5D-3L, VAS (0–100)	53.77 (20.99)
PainDETECT-questionnaire score > 18	16 (16%)
PainDETECT-questionnaire score 13–18	27 (26%)
PainDETECT-questionnaire score < 13	59 (58%)
Pain drawing, pixels	5,144.72 (4,631.32)
Disc herniation segment	
L1/L2	1 (1%)
L2/L3	5 (5%)
L3/L4	5 (5%)
L4/L5	38 (37%)
L5/S1	53 (52%)

The overall mean pressure pain threshold across segments was 447 kPa and the overall mean heat pain threshold was 39.14 °C (Table 2).

**Table 2:** Quantitative sensory testing of pain thresholds.

Outcome	All *N* = 510^*1*^	L1 *N* = 102^*1*^	L2 *N* = 102^*1*^	L3 *N* = 102^*1*^	L4 *N* = 102^*1*^	L5 *N* = 102^*1*^
Pressure Pain Threshold (kPa)	447 (208)	466 (205)	449 (200)	441 (199)	442 (216)	439 (223)
Heat Pain Threshold (°C)	39.14 (3.20)	38.82 (3.60)	39.28 (2.99)	39.24 (3.03)	39.22 (3.22)	39.16 (3.17)

### Association between distance from the symptomatic level of disc herniation and QST

#### Pressure pain threshold

We fitted a linear mixed model (estimated using REML and nloptwrap optimizer) to predict pressure pain threshold with distance to the symptomatic level of disc herniation and symptomatic level of disc herniation (formula: PPT ~ segment distance + diagnosis segment). The model included patient id as random effect (formula: ~1 | patient_id). The model’s total explanatory power is substantial (conditional R2 = 0.92) and the part related to the fixed effects alone (marginal R2) is of 0.01. The model’s intercept, corresponding to segment distance = 0 and symptomatic level of disc herniation = 0, is at 562.85 (95% CI [359.08, 766.61], t(480) = 5.43, *p* < 0.001). Within this model:

- The effect of segment distance is statistically significant and positive (beta = 6.26, 95% CI [1.92, 10.61], t(480) = 2.83, *p* = 0.005; Std. beta = 0.04, 95% CI [0.01, 0.06])
- The effect of diagnosis segment is statistically non-significant and negative (beta = −29.12, 95% CI [−75.27, 17.03], t(480) = −1.24, *p* = 0.216; Std. beta = −0.12, 95% CI [−0.31, 0.07])

**Fig. 2:**
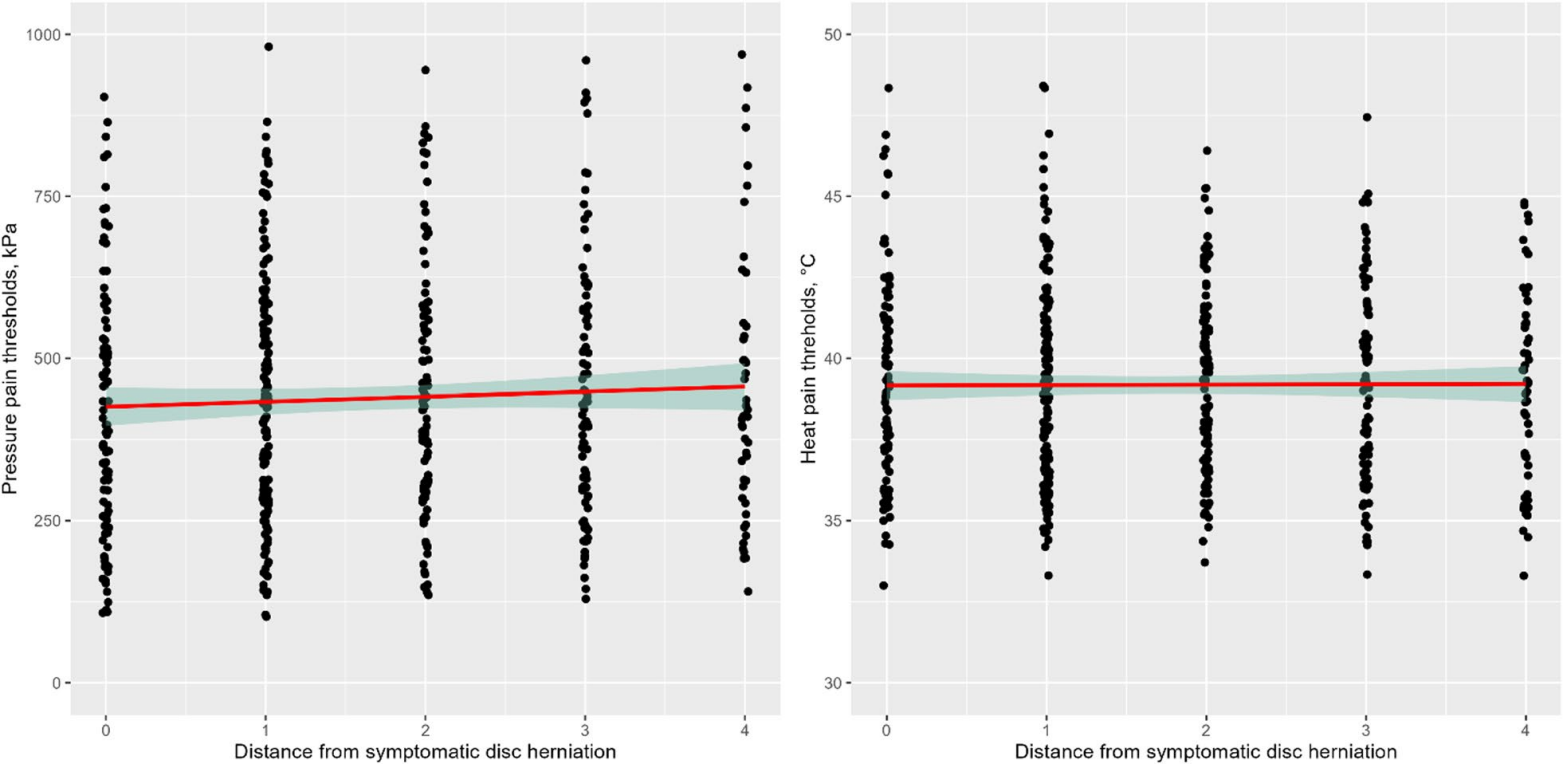
The association between the distance from the symptomatic level of disc herniation and pressure pain thresholds (left panel) and heat pain thresholds (right panel).

### Heat pain threshold

We fitted a linear mixed model (estimated using REML and nloptwrap optimizer) to predict heat pain threshold with distance to the symptomatic level of disc herniation and symptomatic level of disc herniation (formula: HPT ~ segment distance + diagnosis segment). The model included patient id as random effect (formula: ~1 | patient id). The model’s total explanatory power is substantial (conditional R2 = 0.84) and the part related to the fixed effects alone (marginal R2) is of 4.20e-03. The model’s intercept, corresponding to segment distance = 0 and diagnosis segment = 0, is at 38.22 (95% CI [35.07, 41.38], t(500) = 23.80, *p* < 0.001). Within this model:

- The effect of segment distance is statistically non-significant and negative (beta = −0.08, 95% CI [−0.17, 7.93e-03], t(500) = −1.79, *p* = 0.074; Std. beta = −0.03, 95% CI [−0.07, 3.17e-03])
- The effect of diagnosis segment is statistically non-significant and positive (beta = 0.24, 95% CI [−0.47, 0.95], t(500) = 0.67, *p* = 0.503; Std. beta = 0.06, 95% CI [−0.12, 0.25])

Standardized parameters were obtained by fitting the models on a standardized version of the dataset. 95% Confidence Intervals (CIs) and p-values were computed using a Wald t-distribution approximation (Fig. 2).

## Discussion

This study demonstrated a statistically significant association between the paraspinal pressure pain threshold at a lumbar segment and the proximity to that segment to the symptomatic level of disc herniation. No such association was observed for superficial heat pain threshold. The hypothesis of higher pain sensitivity closer to the level of disc herniation was thus confirmed for deep mechanical pressure stimuli, but not for superficial thermal stimuli.

At first glance, it may seem almost self-evident that experimental pain sensitivity is higher closer to the site/segment of clinical pain, given what is known about secondary hyperalgesia, i.e. the increased sensitivity that develops in an area surrounding nociceptive injury^22,23^.

The current findings are noteworthy though, not just for demonstrating a spatial relationship between pain sensitivity and the site of disk herniation/protrusion in individuals with clinical pain, but for the distinct manner of the association: The relationship was evident with mechanical stimulation of deep musculoskeletal structures (muscles), but not for superficial heat stimulation. Ruiz-Ruiz et al. reported a similar observation in lateral epicondylalgia, where the tested muscle was sensitive to pressure but not to thermal stimuli^24^. They suggested this may reflect descending facilitation rather than the peripheral sensitization from the muscle reflecting the PPT. This underlines that the stimulus modality may be important for underlying pain mechanisms, with different modalities reflecting activation of different populations of neurons and pain pathways^25^.

Our findings contrast with other studies which reported no segmental differences in PPT measurements in participants with low back pain^26,27^, but those studies included participants with chronic non-specific low back pain. In pain free individuals Keating et al. found regional differences in spinal PPT values, with the highest PPT values in the lumbar region compared to the thoracic and cervical region^28^. Binderup et al. also published a study on pain free individuals and found no effect on PPT when looking at anatomical subdivisions in the lumbar region^29^. The divergent conclusions suggest that no clear relationship exist between PPT and more caudal spinal test sites. Arguably, this study suggests that well-localized secondary hyperalgesia is feature in LBP from disc herniation, but not necessarily in non-specific LBP.

Although this study found a statistically significant correlation between the para-spinal pressure pain threshold and the proximity to the disc herniation level, the effect observed was minor. Walton et al. reported minimal detectable changes values for PPT at the tibialis anterior and upper trapezius muscles, with values of 47.2 kPa and 97.9 kPa, respectively, in symptomatic participants^30^. These findings are similar to those reported by Koh et al.^31^. Albeit statistically significant for PPT in our study, it seems clear, that the observed differences between segments are too small to be clinically useful. In other words, our findings lend some support to the notion that the affected segment is hyperalgesic compared to adjacent and more distant segments, but the differences are too small to be clinically useful. Evaluation and decision-making in the clinic based solely on pressure pain threshold measurements using the algometer is not warranted on the basis of these results. Never-the-less, the current findings may have clinical implications in so far as many clinicians will use segmental tests such as manual pressure application, springing tests and spinal percussion tests in order to elicit pain responses. The current data lends credence to such clinical procedures as adding diagnostic information by identifying or confirming increased segmental pain sensitivity and by extension, hyperalgesia and nociceptive activity.

### Limitations

Limitations of this study include that we did not test inter-examiner reliability of the QST tests. This has been done in other studies showing high and excellent inter-examiner reliability for the PPT^32^ and good or excellent inter-examiner reliability for the HPT^33^.

Also, practical feasibility dictated that not all tests were conducted by the same rater. This would result in some degree of inter-rater variability, but this has previously been reported not to contribute much towards overall variability^34^.

In the present study we made the assumption that the structurally most affected nerve segment on MRI was in fact the source of nociception associated with symptoms of disk herniation. However, as mentioned in the Background, there is some discussion about the relevance of MRI findings such as disc herniation/protrusion for clinical pain and there is a degree of natural biological variation in the anatomy of neural segmental innervation which may complicate the matter further. Thus it should be borne in mind when interpreting these results, that the *diagnosed segment* may in fact not be the source of clinical pain. Furthermore, the spatial sensory resolution of the lower back is generally quite poor, and especially so in patients with LBP compared to healthy controls^35^.

This study thus involved a well-defined patient group, in which the location of the origin of nociception was, at the very least, believed to be known. However, this limits generalizability to other low back pain patient populations. Future research could explore other spine-related conditions, such as spinal stenosis, spondylolisthesis, compression fractures and other well-localized conditions to assess whether QST responses are similarly related to structural pathology.

This study did not include a healthy control-group. This would have provided insights into the properties of the PPT and HPT in pain-free participants, and notably whether MRI findings in healthy participants were in any way related to such QST.

The segmental distribution of disc herniations in the current population was as might be expected compared to that generally observed in clinical settings, i.e. the majority were seen in the lower two segments. In other words, there was limited variation in the affected segments with only a smaller percentage in the upper lumbar spine. There is a potential risk therefor, that our findings could be confounded by a general tendency for increasing or decreasing deep pressure pain sensitivity further caudally along the spinal column. However, albeit reports on segmental variation in deep pressure pain sensitivity are not entirely aligned, it seems pressure pain threshold typically *increase* in the caudal direction^29^. It seems unlike therefore that our findings are confounded by a general tendency for segmental differences in pressure pain sensitivity in the caudal direction.

Finally, it should be noted, that the analyses did not adjust for potential confounders, such as age and sex, but as we were interested primarily in studying associations between different segments *within* individuals, this is not likely to have affected our results.

In conclusion, the findings support the possible presence of secondary hyperalgesia to deep mechanical pressure in participants with lumbar disc herniation and this is evident as a correlation between pain sensitivity and segmental distance to the herniation. This may have implications in relation to clinical assessments of such patients, especially when there is uncertainty about which segment is causing the symptoms.

## Supplementary Information

Below is the link to the electronic supplementary material.

Supplementary Material 1

Supplementary Material 2

Supplementary Material 3

Supplementary Material 4

## Data Availability

All data supporting the findings of this study are available from the corresponding author upon request.
