# Supplementary material for: Association between pain sensitivity and distance from the affected level of disc herniation
Source: Sci Rep. 2025 Nov 28;15:42893. doi: 10.1038/s41598-025-24918-4 (PMC12669639; doi:10.1038/s41598-025-24918-4)
Supplement: Supplementary file 2 — Supplementary Material 2 [file 41598_2025_24918_MOESM2_ESM.pdf]

Association between pain sensitivity and distance from segmental level of disc herniation: A cross-sectional study of patients with lumbar disc herniation

Trial registration number: clinicaltrials.gov (ID: NCT03832036)

Study protocol version: Version 1.0 – 2018

SAP version number with date: Version 1.0 – 27/06/2023

SAP revision history, justifications, and timing: SAP version 1

1. STUDY OVERVIEW

Background and rationale

A frequent cause of acute low back pain with radiating leg pain is lumbar disc herniation (LDH) [1]. The diagnosis of lumbar disc herniation with radiculopathy is based on history and physical examination. Muscle testing, sensory testing, test of reflexes, straight leg raising test, Lasegue sign and crossed straight leg raising tests are commonly used. To confirm the diagnosis or in cases of clinical disagreement Magnetic Resonance Imaging (MRI) is recommended [1, 2]. However, intervertebral disc herniations and protrusions are also frequently found on MRI in asymptomatic patients [3, 4].

Quantitative Sensory Pain Testing (QST) is a well-defined psychophysical method that standardizes a potentially painful stimulus and quantifies the pain response from the test subject, making it possible to investigate pain processing [5, 6].

Only few studies have investigated the clinical value of (QST) responses in patients with low back pain [7] and it has not yet been shown if the pain sensitivity is different at the level of disc herniation.

Objectives

The aim of this study is to investigate whether the segmental level of disc herniation correlates with pain thresholds in patients with lumbar disc herniation. The hypothesis is that the lowest pain threshold is at the affected level.
The specific objective is to investigate whether patients are sensitized at the level of disc herniation when examined by quantitative sensory testing, in terms of pressure pain and heat pain sensitivity.

Methods

The data from the present study were part of a prospective, 4-year observational study of patients with sciatica due to lumbar disc herniation. This study will be based on data from the baseline examination.

PICOTS

***Population**:*

Patients were consecutively recruited between November 2018 and January 2021 from three different hospitals in Denmark; a) The Spine Center of Southern Denmark at Lillebaelt University Hospital, b) The Department of Neurosurgery at Odense University Hospital and c) The Department of Rheumatology at Frederiksberg Hospital.
Participants were thus all patients recruited in a hospital setting on referral from primary sector.
Inclusion criteria were 18+ years of age and legally competent, fluent in Danish (written and spoken), an MRI-verified disc herniation in the lumbar spine, complained of low back pain radiating below the knee or anterior thigh pain in one or both legs, pain distribution was of a dermatomal pattern, the reported average pain intensity was 3 or more on a Numerical Rating Scale (0-10 NRS), no history of previous spine surgery, surgery in general in the past 4 months, no current use of anticoagulants and/or confounding diagnoses e.g. lumbar spinal stenosis, local muscle trauma, cancer metastases, fibromyalgia and neuropathy.

Exposure:

Distance from segmental level of disc herniation: Clinical and MRI verified symptomatic level of disc herniation is labeled 0, the segmental level above and under is labeled +1 and so forth.

Outcomes:

QST measurements: Pressure Pain Threshold, kPa (PPT) and Heat Pain Threshold, °C (HPT).

Study design:

Observational cross-sectional study.

Further statistical details

Power considerations:

The current sample size had been estimated, based on previous experience and publications in the fields of QST. We had enlisted the assistance of a bio-statistician from the Institute of Regional Health Service Research. Since the project's main objective was to investigate predictor variables, there would be no hypothesis testing, and therefore no statistical basis for a power calculation. The sample size would suffice to demonstrate a statistically significant correlation at a level deemed too also be clinically relevant. The sample size was also in accordance with the previous literature in the field.

Framework:

This is an observational study assessing if patients are sensitized at the level of disc herniation when examined by QST. The data from the present study is part of a prospective, 4-year observational study of patients with sciatica due to lumbar disc herniation.

Timing of final analysis: The statistical analyses are expected to be initiated when this statistical analysis plan has been finalized and signed.

Timing of outcome assessment: (see next section)

Confidence intervals and P values: All 95% confidence intervals and P-values will be two-sided.

Multiplicity: No explicit adjustments but hierarchical testing of primary and secondary analyses as indicated by the order in the outline (Figure 2 is primary).

Statistical software: R version 4.2.2 (or newer).

2. TABULAR PRESENTATION OF TIMING OF OUTCOME MEASUREMENTS

At the first visit, the participants were assessed for eligibility to participate in the study. At this visit, the segmental level of disc herniation was recorded in the participants hospital journals and verified by MRI. Later, the participants were invited to a baseline visit, where all other data for this study was collected.

	**First visit** (assessment for eligibility)	**Second visit** (baseline)
**Variables**		
Segmental level of disc herniation (diagnose)	X	
Pressure Pain Threshold, kPa		X
Thermal Pain Threshold, °C		X
Patient-reported outcomes		X

3. OUTCOMES AND DATA

Data management:

<u>In general for both Pressure Pain Threshold and Thermal Pain Threshold:</u>
Initially, 5 test-sites were marked with a black felt-tip pen in the lumbar region. Patients identified the most painful side (right/left) and the markings were made 2 cm lateral of the spinous process of L1-L5 identified by palpation.

For each stimuli (heat and pressure) we performed the QST tests twice at each test-site. The average of the two measurements at each test-site were used for further analysis. The test order of lumbar test sites was determined a-priori as a computer-generated randomized order for each participant.

Pressure Pain Threshold:
Pressure was applied manually with a Somedic algometer type II (Hörby, Sweden, 1 cm2 probe) on the marked test-sites, with a near-constant increase in pressure of 50 kPa/s until the participant indicated the pressure becoming painful by pressing an indicator button connected to the algometer.

Thermal Pain Threshold:
With a Medoc TSA-II thermode stimulator the heat pain threshold was measured on the marked test sites. The thermode stimulator had a baseline temperature of 32°C and increased 1°C/s. The patient indicated when the stimulus was perceived as becoming painful by pressing an indicator button, upon which the temperature returned to baseline (decrease 10°C/s).

Patient-Reported Outcomes:

Age
Sex
Height
Weight
Back pain intensity
Leg pain intensity
Previous pain
Use of pain medication
Physical activity
Alcohol status
Work status
Sick leave

Roland-Morris Questionnaire: Assessment of physical disability due to low-back pain.
EQ-5D Questionnaire: Assessment of health-related quality of life.
PainDetect Questionnaire: Assessment of pain and neuropathic components.

Pain drawing: A pain drawing, was filled out by pen-on-paper. This was transformed to a digital PDF, where the total area of pain was calculated as number of pixels.

Segmental level of disc herniation (diagnose)
As recorded in the participants hospital journal and verified by MRI.

Data validation:
All variables used in the analyses, will be checked for missing values, outliers and inconsistencies.

4. PROTOCOL DEVIATIONS WITH BEARING ON THIS STATISTICAL ANALYSIS PLAN

The following details in this SAP represent deviations from the trial protocol.

Header in the protocol	Change	Reason
(currently no deviations)		

5. OUTLINE

The anticipated (predefined) outline of the manuscript is illustrated below.

Results that only will be presented in the manuscript text include:
- Number of participants included from Frederiksberg, Odense and Middelfart, respectively.
- Days on sick leave among those currently on sick leave.
- Duration of pain medication intake in days among those currently using pain medication.
- Further detailed results from the regression models visualized in figure 2 and 3.

Figure 1. Flow diagram
Anticipated plot design, illustrating potential reasons for exclusion:

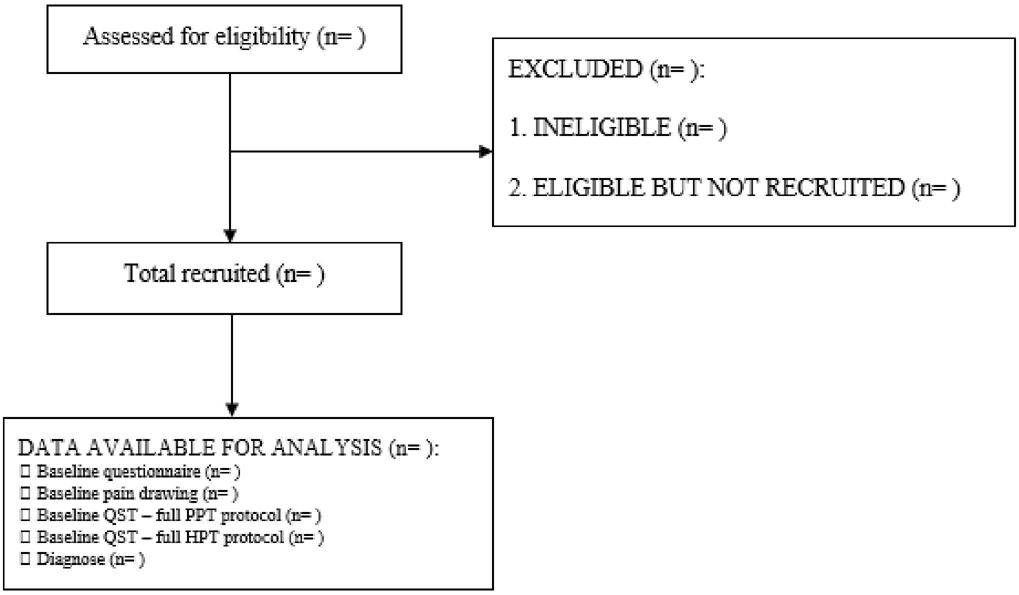

Table 1. Patient characteristics

Characteristics	Total (n=)
Demographics	
Age, years	
Female, n(%)	
Body Mass Index, kg/m^2	
Back pain intensity, NRS (0-10)	
Leg pain intensity, NRS (0-10)	
Previous pain, n(%)	
Use of pain medication, n(%)	
Physical activity:	
Inactive, n(%)	
Low-impact physical activity ≥ 4 hours/week, n(%)	
High-impact physical activity ≥ 3 hours/week, n(%)	
Don’t know, n(%)	
Smoking status:	
No, I don't smoke cigarettes, n(%)	
Yes, I smoke cigarettes only occasionally, n(%)	
Yes, I smoke 1 to 4 cigarettes per day, n(%)	
Yes, I smoke 5 to 14 cigarettes per day, n(%)	
Yes, I smoke 15 to 24 cigarettes per day, n(%)	
Yes, I smoke 25 or more cigarettes per day, n(%)	
Don’t know, n(%)	
Alcohol status, units per week:	
0, n(%)	
1-7, n(%)	
8-14, n(%)	
15 or more, n(%)	
Don't know, n(%)	
Work status:	
Employed (eg. ordinary job, studying), n(%)	
Unemployed (eg. Senior or early retirement pension), n(%)	
Don’t know, n(%)	
Sick leave, n(%)	
Questionnaires	
Roland-Morris Questionnaire	
EQ5D-3L, index score	
EQ5D-3L, VAS (0-100)	
PainDetect Questionnaire:	
Score ≥ 18, n(%)	
Score 13-18, n(%)	
Score <13, n(%)	
Pain drawing	
Pain drawing, pixels	
Segmental level of disc herniation (diagnose)	
L1, n(%)	
L2, n(%)	
L3, n(%)	
L4, n(%)	
L5, n(%)	

Further statistical information related to table 1:
Data will be presented as mean with standard deviation (SD) when normally distributed or as median with interquartile range in case of non-parametric data. Dichotomous and categorical data will be presented in proportions and/or frequency/count. Normality of the data will be assessed using Q-Q plots, and histograms.

Table 2. Quantitative Sensory Testing outcome measurents

	Segment	n	Mean (SD)
**Outcome**			
Pressure Pain Threshold	All		
	L1		
	L2		
	L3		
	L4		
	L5		
Heat Pain Threshold	All		
	L1		
	L2		
	L3		
	L4		
	L5		

Further statistical information related to table 2:
Data will be presented as mean with standard deviation (SD) when normally distributed or as median with interquartile range in case of non-parametric data. Normality of the data will be assessed using Q-Q plots, and histograms.

Figure 2: The association between the distance from the segmental level of disc herniation and pressure pain sensitivity (panel A) and heat pain sensitivity (panel B).

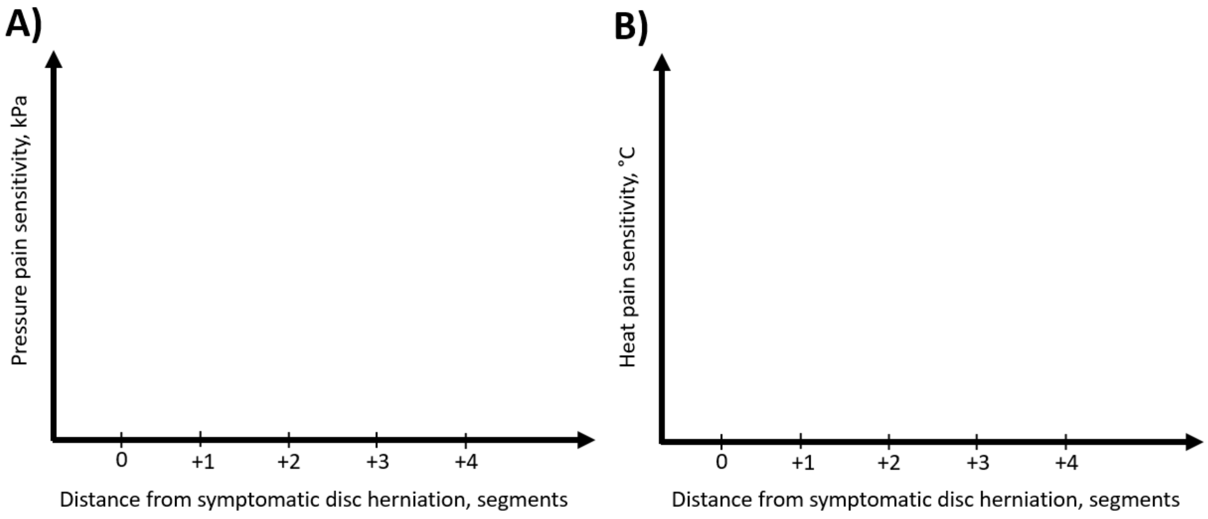

Values are least-squares means from a linear mixed models including the participant as random effect, distance from symptomatic disc herniation (5 levels; 0, 1, 2, 3, 4 segments) as fixed effect, as well as the value at 0 segments as covariate. Missing data for a specific distance will be handled implicitly by the repeated measures mixed linear models, assuming data missing at random (MAR). Error bars will indicate standard errors.

Assumptions will be checked by visual inspection of residual plots assessing the normality of residuals. In case the distributional assumptions do not hold, we will use an appropriate transformation (e.g., log-transformation in the case of right-skewed data and report the results as geometric means and geometric mean ratios), and/or, use non-parametric methods.

Figure 3: The association between the distance from the segmental level of disc herniation and pressure pain sensitivity (panel A) and heat pain sensitivity (panel B), stratified by site of symptomatic disc herniation.

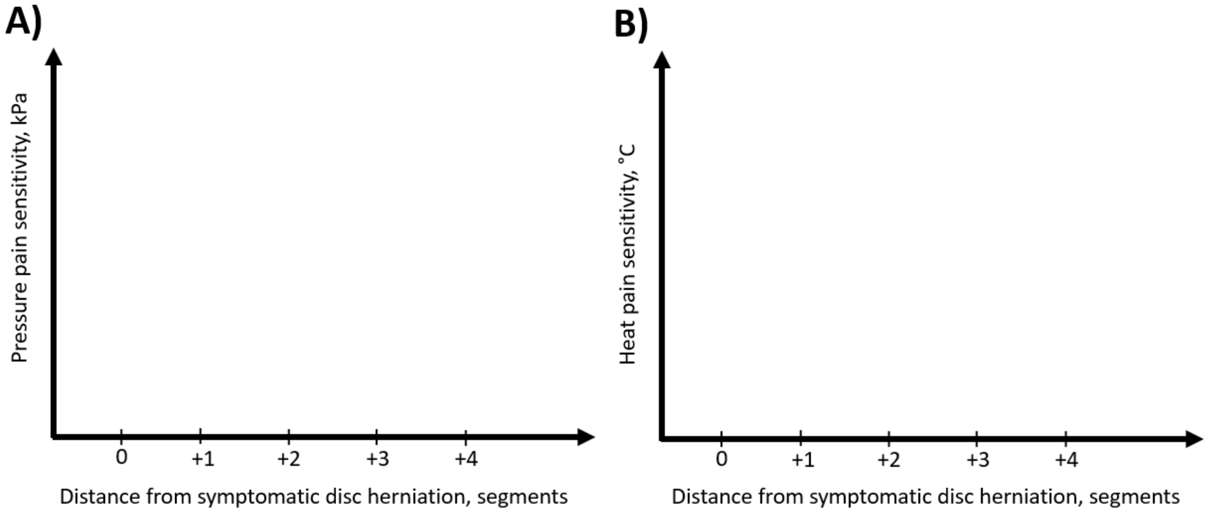

Values are least-squares means from a linear mixed models including the participant as random effect, distance from symptomatic disc herniation (5 levels; 0, 1, 2, 3, 4 segments) and site of symptomatic disc herniation (5 levels, L1, L2, L3, L4, L5) as fixed effects, their interaction, as well as the value at 0 segments as covariate. Missing data for a specific distance will be handled implicitly by the repeated measures mixed linear models, assuming data missing at random (MAR).
Error bars will indicate standard errors.

SUPPLEMENTARY MATERIAL

The anticipated (predefined) supplementary material of the manuscript is illustrated below.

Supplementary file 1. This SAP

Supplementary Table 1. Patient characteristics by site of symptomatic disc herniation

**Characteristics**	**L1 (n=)**	**L2 (n=)**	**L3 (n=)**	**L4 (n=)**	**L5 (n=)**
Demographics					
Age, years					
Female, n(%)					
Body Mass Index, kg/m^2					
(…etc. as shown in Table 1)					

In case of small number of participants for some sites of symptomatic disc herniation, these will be merged with a neighbouring site (e.g., merging L4 and L5).

6. REFERENCES

1. Kreiner DS, Hwang SW, Easa JE, Resnick DK, Baisden JL, Bess S, m.fl. An evidence-based clinical guideline for the diagnosis and treatment of lumbar disc herniation with radiculopathy. The Spine Journal. 2014;14(1):180–91.

2. Van Der Windt DA, Simons E, Riphagen II, Ammendolia C, Verhagen AP, Laslett M, m.fl. Physical examination for lumbar radiculopathy due to disc herniation in patients with low-back pain. The Cochrane Library. 2010;

3. Kjaer P, Leboeuf-Yde C, Korsholm L, Sorensen JS, Bendix T. Magnetic resonance imaging and low back pain in adults: a diagnostic imaging study of 40-year-old men and women. Spine. 2005;30(10):1173–80.

4. Boden SD, Davis D, Dina T, Patronas N, Wiesel S. Abnormal magnetic-resonance scans of the lumbar spine in asymptomatic subjects. A prospective investigation. J Bone Joint Surg Am. 1990;72(3):403–8.

5. Chong PST, Cros DP. Technology literature review: quantitative sensory testing. Muscle & nerve. 2004;29(5):734–47.

6. O'Neill S, Manniche C, Graven-Nielsen T, Arendt-Nielsen L. Association between a composite score of pain sensitivity and clinical parameters in low-back pain. The Clinical journal of pain. 2014;30(10):831–8.

7. Marcuzzi A, Dean CM, Wrigley PJ, Chakiath RJ, Hush JM. Prognostic value of quantitative sensory testing in low back pain: a systematic review of the literature. Journal of Pain Research. 2016;9:599.

8. Hiemstra B, Keus F, Wetterslev J, Gluud C, van der Horst ICC. DEBATE-statistical analysis plans for observational studies. BMC Medical Research Methodology. 9. december 2019;19(1):233.

9. Gamble C, Krishan A, Stocken D, Lewis S, Juszczak E, Doré C, m.fl. Guidelines for the Content of Statistical Analysis Plans in Clinical Trials. JAMA. 19. december 2017;318(23):2337–43.

7. SAP REPORTING GUIDELINE

This SAP has been reported according to the items recommended by the DEBATE guideline for statistical analysis plans for observational studies [8] adapted from Gamble et al. [9].

Table A. Recommended content of statistical analysis plans for observational studies

Section/Item	Index	Description	Location in this SAP
**Section 1: Administrative Information**			
Title and trial registration	1a	Descriptive title that matches the protocol, with SAP either as a forerunner or subtitle, and study acronym	*Front page*
	1b	Study registration number	*Front page*
SAP version	2	SAP version number with dates	*Front page*
Protocol version	3	Reference to version of protocol being used	*Front page*
SAP revisions	4a	SAP revision history	*Front page*
	4b	Justification for each SAP revision	*Front page*
	4c	Timing of SAP revisions in relation to planned repetitive analyses	*Front page*
Roles and responsibility	5	Names, affiliations, and roles of SAP contributors	*Front page*
Signatures of:	6a	Person writing the SAP	*Front page*
	6b	Senior statistician responsible	*Front page*
	6c	Chief investigator/clinical lead	*Front page*
**Section 2: Introduction**			
Background and rationale	7	Synopsis of study background and rationale including a brief description of research question and brief justification for undertaking the study	*1. Study overview*
Objectives	8	Description of specific objectives and hypotheses, including secondary objectives	*1. Study overview*
**Section 3: Study Methods**			
Study design	9	Brief description of study design including type of study (e.g. case-control, cross-sectional or cohort study)	*1. Study overview*
Power considerations	10	In case of an unspecified sample size, provide power calculations for (at least) the primary analysis or present a detectable difference with a specified power	*1. Study overview*
Framework	11	Superiority, equivalence, or noninferiority hypothesis testing framework, including which comparisons will be presented on this basis	*1. Study overview*
Statistical interim analyses and stopping guidance	12a	Information on repetitive analyses specifying what repetitive analyses will be carried out and listing of time points	*1. Study overview*
	12b	Any planned adjustment of the significance level due to repetitive analyses	*1. Study overview*
	12c	Details of guidelines for stopping the study early	-
Timing of final analysis	13	Timing of final analysis, e.g., all outcomes analysed collectively or timing stratified by planned length of follow-up	-
Timing of outcome assessments	14	Time points at which the outcomes are measured including visit "windows"	*2. Tabular presentation of timing of outcome measurements*
**Section 4: Statistical Principles**			
Confidence intervals and *P* values	15	Level of statistical significance	*1. Study overview*
	16	Description and rationale for any adjustment for multiplicity and, if so, detailing how the type 1 error is to be controlled	*1. Study overview*
	17	Confidence intervals to be reported	*1. Study overview*
Adherence and protocol deviations	18a	Definition of protocol deviations for the study	*4. Protocol deviations with bearing on this statistical analysis plan*
	18b	Description of which protocol deviations will be summarized	*4. Protocol deviations with bearing on this statistical analysis plan*
Analysis populations	19	Definition of analysis populations, eg, intention to treat, per protocol, complete case, safety	*5. Outline (for each table and figure)*

**Section 5: Study Population**			
Screening data	20	Reporting of screening data (if collected) to describe representativeness of trial sample	-
Eligibility	21	Summary of eligibility criteria	*1. Study overview*
Recruitment	22	Information to be included in the STROBE flow diagram	*5. Outline (Figure 1)*
Withdrawal/follow-up	23a	Level of withdrawal, e.g., dropouts after inclusion or refusal to be contacted for additional information	*5. Outline (Figure 1)*
	23b	Timing of withdrawal/lost to follow-up data	*5. Outline (Figure 1)*
	23c	Reasons and details of how withdrawal/lost to follow-up data will be presented	*5. Outline (Figure 1)*
Baseline patient characteristics	24a	List of baseline characteristics to be summarized	*5. Outline (Table 1)*
	24b	Details of how baseline characteristics will be descriptively summarized	*5. Outline (Table 1)*
Potential confounding covariates	25	A description of potential confounding covariates and how these will be dealt with	-
**Section 6: Analysis**			
Outcome definitions		List and describe each primary and secondary outcome including details of:	
	26a	Specification of outcomes and timings. If applicable include the order of importance of primary or key secondary endpoints (e.g., order in which they will be tested)	*5. Outline (mainly Table 2)*
	26b	Specific measurement and units (e.g., glucose control, HbA1c [mmol/mol or %])	*5. Outline (Table 2)*
	26c	Any calculation or transformation used to derive the outcome (e.g., change from baseline, QoL score, time to event, logarithm, etc)	*5. Outline, and 3. Outcomes and data*
Analysis methods	27a	What analysis method will be used and how the treatment effects will be presented	*5. Outline*
	27b	Any adjustment for covariates	*5. Outline*
	27c	Methods used for assumptions to be checked for statistical methods	*5. Outline*
	27d	Details of alternative methods to be used if distributional assumptions do not hold, eg, normality, proportional hazards, etc	*5. Outline*
	27e	Any planned sensitivity analyses for each outcome where applicable	*5. Outline*
	27f	Any planned subgroup analyses for each outcome including how subgroups are defined	*5. Outline*
Missing data	28	Reporting and assumptions/statistical methods to handle missing data (e.g., multiple imputation)	*5. Outline*
Additional analyses	29	Details of any additional statistical analyses required, eg, complier-average causal effect analysis	*5. Outline*
Harms	30	Only applies when intervention effects are studied. Sufficient detail on summarizing safety data, e.g. information on severity, expectedness, and associations; details of how adverse events are scored; how adverse event data will be analysed and the follow-up time.	-
Statistical software	31	Details of statistical packages to be used to carry out analyses	*1. Study overview*
References	32a	References to be provided for nonstandard statistical methods	*6. References*
	32b	Reference to Data Management Plan	-
	32c	Reference to the Study Master File and Statistical Master File	-
	32d	Reference to other standard operating procedures or documents to be adhered to	-

Abbreviations: STROBE, STrengthening the Reporting of OBservational studies in Epidemiology; HbA1c, hemoglobin A1c; QoL, quality of life; SAP, statistical analysis plan.

Supplementary File 3 Subgrouped analysis of HPT and PPT.

Pressure pain threshold

We fitted a linear mixed model (estimated using REML and nloptwrap optimizer) to predict pressure pain thresholds with distance to the segmental level of disc herniation and segmental level of disc herniation (formula: PPT ~ segment_distance * factor(diagnosis segment) + diagnosis segment). The model included the patient id as random effect (formula: ~1 | patient id). The model's total explanatory power is substantial (conditional R2 = 0.92) and the part related to the fixed effects alone (marginal R2) is of 0.05. The model's intercept, corresponding to distance to the segmental level of disc herniation = 0 and segmental level of disc herniation = 0, is at 887.20 (95% CI [486.69, 1287.71], t(473) = 4.35, p < .001). Within this model:

- The effect of segment distance is statistically non-significant and positive (beta = 4.10, 95% CI [-33.23, 41.43], t(473) = 0.22, p = 0.829; Std. beta = 0.03, 95% CI [-0.20, 0.25])
- The effect of diagnosis segment [2] is statistically significant and negative (beta = -470.36, 95% CI [-876.39, -64.33], t(473) = -2.28, p = 0.023; Std. beta = -1.97, 95% CI [-4.04, 0.11])
- The effect of diagnosis segment [3] is statistically significant and negative (beta = -454.97, 95% CI [-859.47, -50.47], t(473) = -2.21, p = 0.028; Std. beta = -2.07, 95% CI [-4.14, 1.22e-03])
- The effect of diagnosis segment [4] is statistically non-significant and negative (beta = -380.53, 95% CI [-819.20, 58.13], t(473) = -1.70, p = 0.089; Std. beta = -2.21, 95% CI [-4.12, -0.29])
- The effect of diagnosis segment [5] is statistically non-significant and negative (beta = -416.54, 95% CI [-855.64, 22.56], t(473) = -1.86, p = 0.063; Std. beta = -2.17, 95% CI [-4.08, -0.26])
- The effect of segment distance × diagnosis segment [2] is statistically non-significant and positive (beta = 6.35, 95% CI [-31.96, 44.66], t(473) = 0.33, p = 0.745; Std. beta = -0.10, 95% CI [-0.37, 0.17])
- The effect of segment distance × diagnosis segment [3] is statistically non-significant and positive (beta = 1.81, 95% CI [-35.89, 39.50], t(473) = 0.09, p = 0.925; Std. beta = -0.05, 95% CI [-0.35, 0.25])
- The effect of segment distance × diagnosis segment [4] is statistically non-significant and negative (beta = -16.80, 95% CI [-

60.73, 27.12], t(473) = -0.75, p = 0.453; Std. beta = 0.04, 95% CI [-0.20, 0.27])
- The effect of segment distance × diagnosis segment [5] is statistically non-significant and negative (beta = -8.61, 95% CI [-57.49, 40.26], t(473) = -0.35, p = 0.729; Std. beta = 0.01, 95% CI [-0.22, 0.24])

Standardized parameters were obtained by fitting the models on a standardized version of the dataset. 95% Confidence Intervals (CIs) and p-values were computed using a Wald t-distribution approximation (Figure 3).

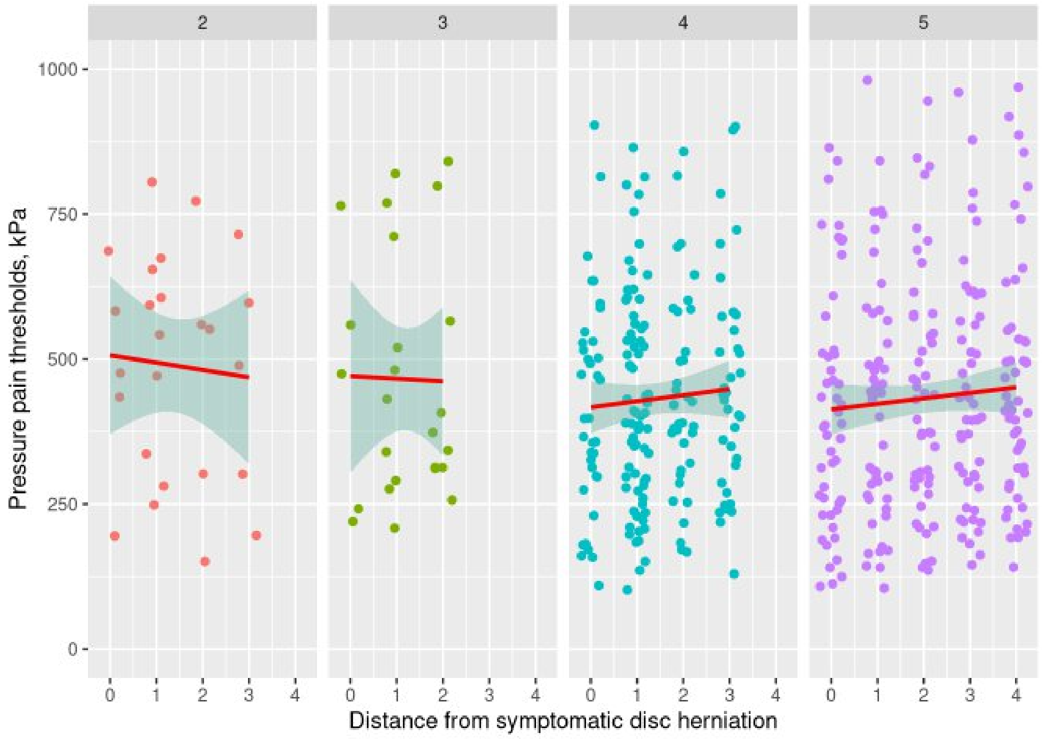

Figure 3: The association between the distance from the segmental level of disc herniation and pressure pain sensitivity when stratified by level of disc herniation (2-5).

Heat pain threshold

We fitted a linear mixed model (estimated using REML and nloptwrap optimizer) to predict heat pain thresholds with segmental level of disc herniation and segmental level of disc herniation (formula: HPT ~ segment distance * factor(diagnosis segment) + diagnosis segment). The model included patient id as random effect (formula: ~1 | patient id). The

model’s total explanatory power is substantial (conditional R2 = 0.85) and the part related to the fixed effects alone (marginal R2) is of 0.03. The model’s intercept, corresponding to the segmental level of disc herniation = 0 and segmental level of disc herniation = 0, is at 41.32 (95% CI [35.20, 47.44], t(493) = 13.27, p < .001). Within this model:

- The effect of segment distance is statistically non-significant and positive (beta = 0.47, 95% CI [-0.32, 1.26], t(493) = 1.17, p = 0.243; Std. beta = 0.19, 95% CI [-0.13, 0.50])
- The effect of diagnosis segment [2] is statistically non-significant and negative (beta = -2.00, 95% CI [-8.20, 4.20], t(493) = -0.63, p = 0.526; Std. beta = -1.69, 95% CI [-3.76, 0.37])
- The effect of diagnosis segment [3] is statistically non-significant and negative (beta = -1.90, 95% CI [-8.08, 4.27], t(493) = -0.60, p = 0.546; Std. beta = -1.01, 95% CI [-3.03, 1.02])
- The effect of diagnosis segment [4] is statistically non-significant and negative (beta = -4.06, 95% CI [-10.89, 2.78], t(493) = -1.17, p = 0.244; Std. beta = -0.91, 95% CI [-2.78, 0.97])
- The effect of diagnosis segment [5] is statistically non-significant and negative (beta = -2.73, 95% CI [-9.44, 3.98], t(493) = -0.80, p = 0.424; Std. beta = -0.90, 95% CI [-2.76, 0.97])
- The effect of segment distance × diagnosis segment [2] is statistically non-significant and negative (beta = -0.53, 95% CI [-1.34, 0.28], t(493) = -1.28, p = 0.201; Std. beta = -0.32, 95% CI [-0.70, 0.07])
- The effect of segment distance × diagnosis segment [3] is statistically non-significant and negative (beta = -0.57, 95% CI [-1.37, 0.23], t(493) = -1.40, p = 0.162; Std. beta = -0.11, 95% CI [-0.53, 0.30])
- The effect of segment distance × diagnosis segment [4] is statistically non-significant and negative (beta = -0.80, 95% CI [-1.76, 0.17], t(493) = -1.63, p = 0.104; Std. beta = -0.21, 95% CI [-0.53, 0.11])
- The effect of segment distance × diagnosis segment [5] is statistically non-significant and negative (beta = -0.29, 95% CI [-1.32, 0.75], t(493) = -0.55, p = 0.586; Std. beta = -0.23, 95% CI [-0.55, 0.09])

Standardized parameters were obtained by fitting the models on a standardized version of the dataset. 95% Confidence Intervals (CIs) and p-values were computed using a Wald t-distribution approximation (Figure 4).

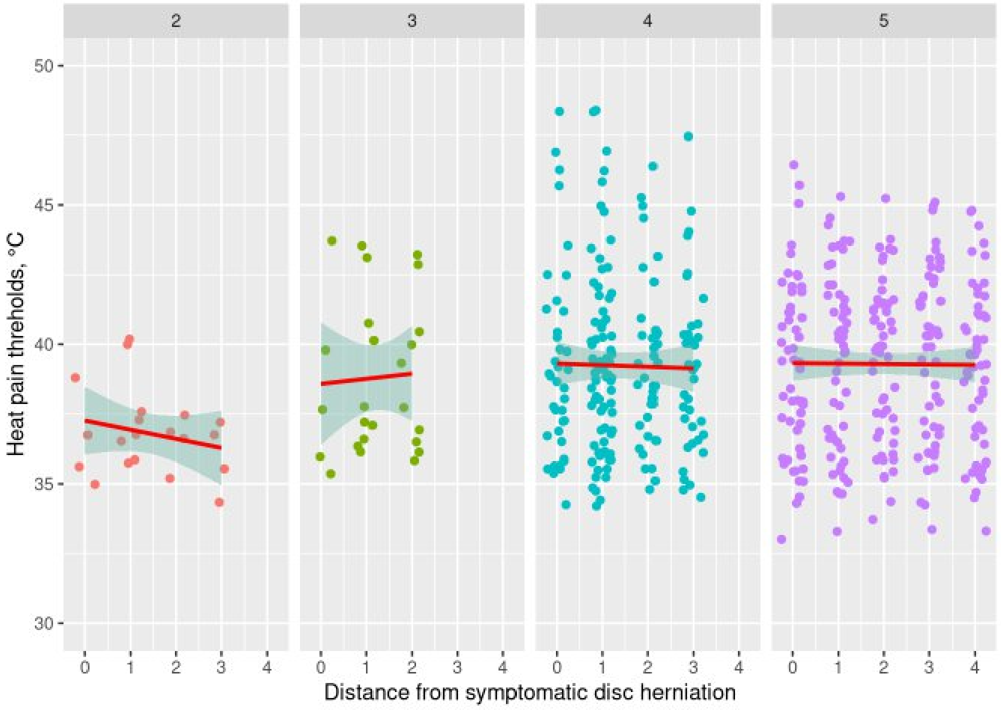

Figure 4: The association between the distance from the segmental level of disc herniation and heat pain sensitivity when stratified by level of disc herniation (2-5).

Supplementary File 4:

Table 3. Patient characteristics by level of disc herniation.

	**L1/L2** N = 1[1]	**L2/L3** N = 5[1]	**L3/L4** N = 5[1]	**L4/L5** N = 38[1]	**L5/S1** N = 53[1]
Age, years	55 (NA)	60 (16)	61 (11)	50 (15)	43 (11)
Sex					
Female	1 (100%)	2 (40%)	3 (60%)	19 (50%)	23 (43%)
Male	0 (0%)	3 (60%)	2 (40%)	19 (50%)	30 (57%)
Body Mass Index, kg/m^2	37.7 (NA)	28.8 (4.7)	24.1 (4.3)	26.9 (5.3)	26.2 (4.3)
Back pain, NRS (0-10)	3 (NA)	5 (3)	5 (2)	4 (3)	5 (2)
Leg pain, NRS (0-10)	3 (NA)	5 (4)	5 (2)	5 (3)	5 (3)
Previous pain					
Yes	1 (100%)	5 (100%)	3 (60%)	29 (76%)	40 (77%)
No	0 (0%)	0 (0%)	2 (40%)	9 (24%)	11 (21%)
Don't know	0 (0%)	0 (0%)	0 (0%)	0 (0%)	1 (2%)
Use of pain medication	0 (0%)	3 (60%)	3 (60%)	28 (74%)	34 (65%)
Duration of pain medication					
Less than 1 week	0 (NA%)	0 (0%)	0 (0%)	1 (4%)	1 (3%)
Between 1 week and 2 months	0 (NA%)	1 (33%)	0 (0%)	9 (32%)	9 (26%)
More than 2 months	0 (NA%)	2 (67%)	2 (67%)	18 (64%)	24 (71%)
Don't know	0 (NA%)	0 (0%)	1 (33%)	0 (0%)	0 (0%)
Physical activity					
Inactive	0 (0%)	1 (20%)	0 (0%)	8 (21%)	5 (10%)
Low-impact physical activity ≥ 4 hours/week	1 (100%)	2 (40%)	3 (60%)	18 (47%)	35 (67%)
High-impact physical activity ≥ 3 hours/week	0 (0%)	1 (20%)	2 (40%)	11 (29%)	10 (19%)
Don't know	0 (0%)	1 (20%)	0 (0%)	1 (3%)	2 (4%)
Smoking status					
No, I don't smoke cigarettes	1 (100%)	5 (100%)	3 (60%)	31 (82%)	32 (62%)
Yes, I smoke cigarettes only occasionally	0 (0%)	0 (0%)	1 (20%)	2 (5%)	10 (19%)
Yes, I smoke 1 to 4 cigarettes per day	0 (0%)	0 (0%)	0 (0%)	1 (3%)	0 (0%)
Yes, I smoke 5 to 14 cigarettes per day	0 (0%)	0 (0%)	1 (20%)	3 (8%)	7 (13%)
Yes - I smoke 15 to 24 cigarettes per day	0 (0%)	0 (0%)	0 (0%)	1 (3%)	2 (4%)
I smoke 25 or more cigarettes per day	0 (0%)	0 (0%)	0 (0%)	0 (0%)	0 (0%)
Don't know	0 (0%)	0 (0%)	0 (0%)	0 (0%)	1 (2%)
Alcohol use					
0	1 (100%)	1 (20%)	2 (40%)	14 (37%)	17 (33%)
1	0 (0%)	2 (40%)	2 (40%)	17 (45%)	26 (50%)
2	0 (0%)	2 (40%)	0 (0%)	4 (11%)	8 (15%)
3	0 (0%)	0 (0%)	1 (20%)	3 (8%)	1 (2%)

Supplementary File 4:

Table 3. Patient characteristics by level of disc herniation.

	**L1/L2** N = 1[1]	**L2/L3** N = 5[1]	**L3/L4** N = 5[1]	**L4/L5** N = 38[1]	**L5/S1** N = 53[1]
Work status					
Employed	1 (100%)	2 (40%)	1 (20%)	26 (68%)	43 (83%)
Unemployed	0 (0%)	3 (60%)	4 (80%)	12 (32%)	9 (17%)
Sick leave					
Yes	0 (0%)	1 (20%)	0 (0%)	12 (32%)	21 (40%)
No	1 (100%)	4 (80%)	5 (100%)	26 (68%)	31 (60%)
Don't want to answer	0 (0%)	0 (0%)	0 (0%)	0 (0%)	0 (0%)
Roland-Morris Questionnaire	74 (NA)	63 (25)	70 (15)	48 (26)	56 (23)
EQ5D-3L, index score	0.66 (NA)	0.64 (0.23)	0.59 (0.19)	0.64 (0.17)	0.65 (0.14)
EQ5D-3L, VAS (0-100)	32 (NA)	61 (31)	45 (14)	56 (22)	53 (20)
PD-Q score ≥ 18	1 (100%)	2 (40%)	0 (0%)	6 (16%)	10 (19%)
PD-Q score 13-18	0 (0%)	1 (20%)	1 (20%)	11 (29%)	14 (26%)
PD-Q score <13	0 (0%)	3 (60%)	5 (100%)	25 (66%)	34 (64%)
Pain drawing, pixels	7,064 (NA)	8,176 (10,976)	5,040 (3,250)	4,939 (3,813)	4,960 (4,457)

[1]Mean (SD); n (%)
