# Supplementary figures and images for: Association between pain sensitivity and distance from the affected level of disc herniation
Source: Sci Rep. 2025 Nov 28;15:42893. doi: 10.1038/s41598-025-24918-4 (PMC12669639; doi:10.1038/s41598-025-24918-4)

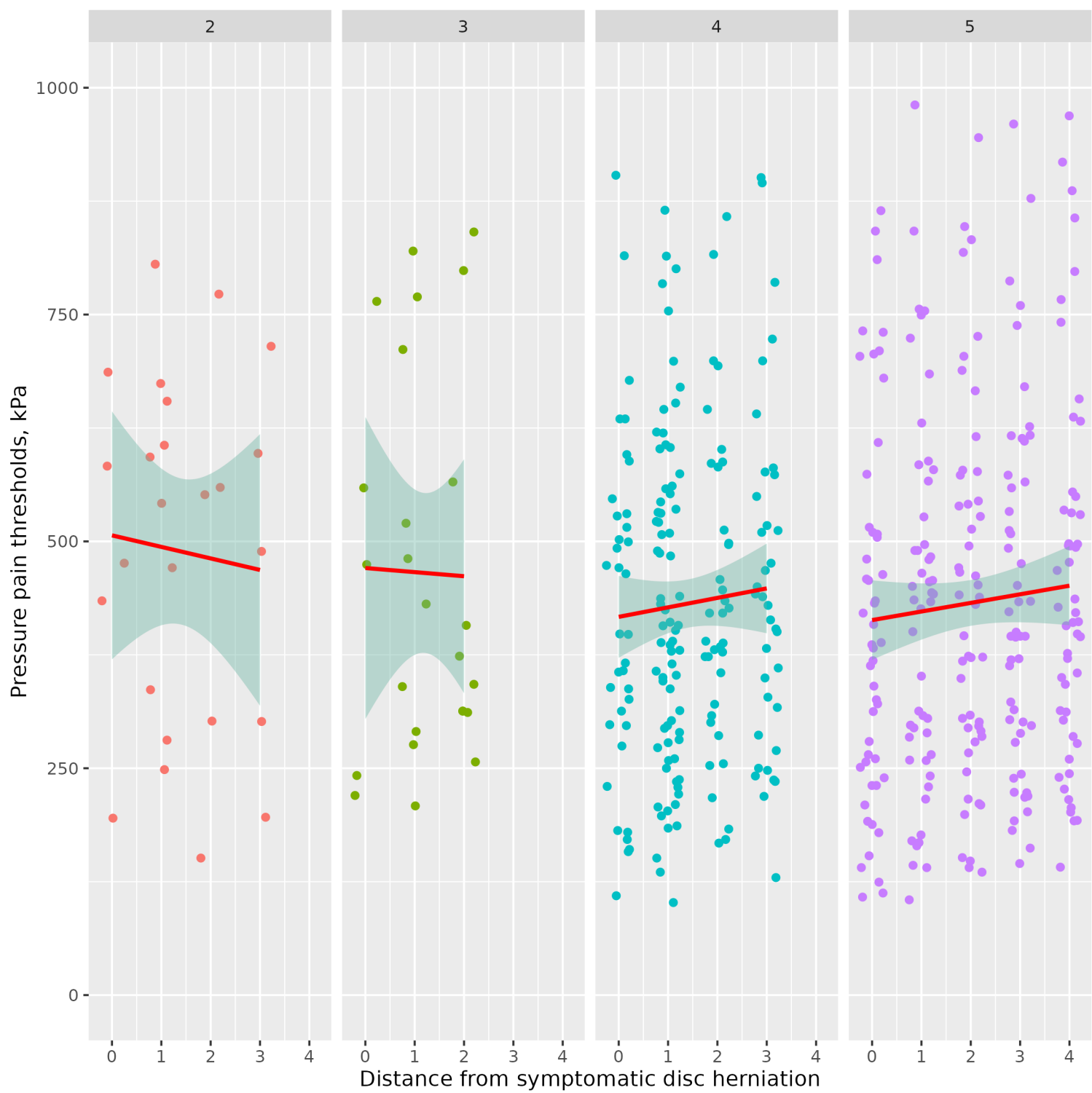

Supplement: Supplementary file 3 — Supplementary Material 3 [file 41598_2025_24918_MOESM3_ESM.pdf]

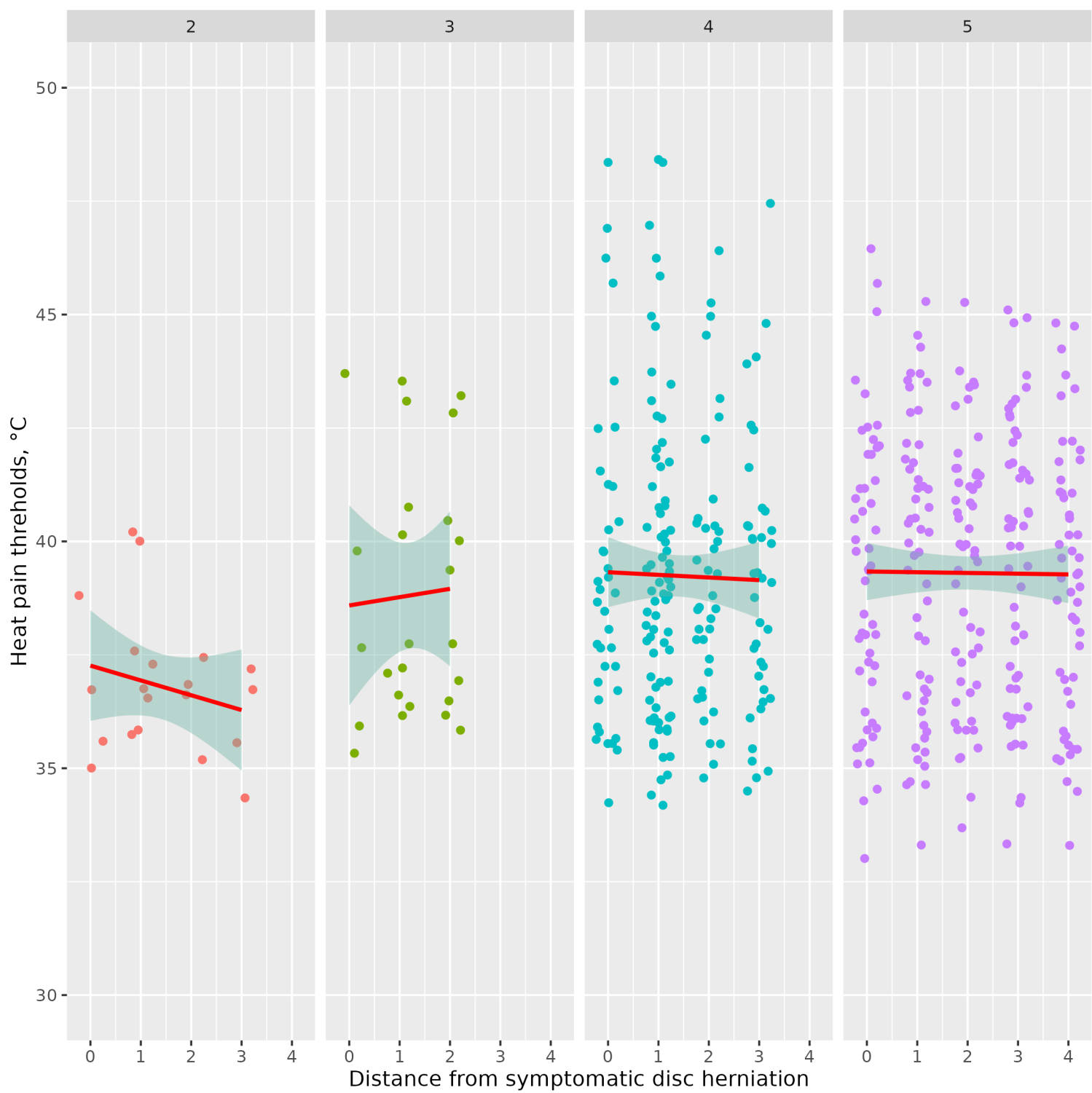

Supplement: Supplementary file 4 — Supplementary Material 4 [file 41598_2025_24918_MOESM4_ESM.pdf]
